# Infective Endocarditis during Pregnancy—Keep It Safe and Simple!

**DOI:** 10.3390/medicina59050939

**Published:** 2023-05-12

**Authors:** Viviana Aursulesei Onofrei, Cristina Andreea Adam, Dragos Traian Marius Marcu, Radu Crisan Dabija, Alexandr Ceasovschih, Mihai Constantin, Elena-Daniela Grigorescu, Antoneta Dacia Petroaie, Florin Mitu

**Affiliations:** 1 Department of Medical Specialties I, II, III and Preventive Medicine and Interdisciplinary, “Grigore T. Popa” University of Medicine and Pharmacy, University Street No. 16, 700115 Iasi, Romania; 2 “St. Spiridon” Clinical Emergency Hospital, Independence Boulevard No. 1, 700111 Iasi, Romania; 3 Cardiovascular Rehabilitation Clinic, Clinical Rehabilitation Hospital, Pantelimon Halipa Street No. 14, 700661 Iasi, Romania; 4 Clinical Hospital of Pneumophthisiology Iași, Doctor Iosif Cihac Street No. 30, 700115 Iasi, Romania; 5 Academy of Medical Sciences, Ion C. Brătianu Boulevard No. 1, 030167 Bucharest, Romania; 6 Academy of Romanian Scientists, Professor Dr. Doc. Dimitrie Mangeron Boulevard No. 433, 700050 Iasi, Romania

**Keywords:** infective endocarditis, pregnancy, cardiovascular maternal risk, multidisciplinary approach, heart team

## Abstract

The diagnosis of infective endocarditis (IE) during pregnancy is accompanied by a poor prognosis for both mother and fetus in the absence of prompt management by multidisciplinary teams. We searched the electronic databases of PubMed, MEDLINE and EMBASE for clinical studies addressing the management of infective endocarditis during pregnancy, with the aim of realizing a literature review ranging from risk factors to diagnostic investigations to optimal therapeutic management for mother and fetus alike. The presence of previous cardiovascular pathologies such as rheumatic heart disease, congenital heart disease, prosthetic valves, hemodialysis, intravenous catheters or immunosuppression are the main risk factors predisposing patients to IE during pregnancy. The identification of modern risk factors such as intracardiac devices and intravenous drug administration as well as genetic diagnostic methods such as cell-free deoxyribonucleic acid (DNA) next-generation sequencing require that these cases be addressed in multidisciplinary teams. Guiding treatment to eradicate infection and protect the fetus simultaneously creates challenges for cardiologists and gynecologists alike.

## 1. Infective Endocarditis during Pregnancy—An Introduction to a Complex Issue

Cardiovascular disease is responsible for complications in 1–4% of pregnancies and 25% of maternal deaths [1]. Infective endocarditis (IE) is a rare condition during pregnancy, with an extremely rare incidence rate of less than 0.01% [2]. From 1893 when the first case of IE was reported by William Bart Osler to the present day, advances in diagnostic methods and the development of new therapeutic molecules have allowed for prompt diagnosis as well as the possibility of quickly administering a targeted antibiotic regimen to reduce the risk of morbidity and mortality for both mother and fetus. The use of multiple imaging assessment methods affords the possibility of surgical intervention during the acute infectious process as well as the development of therapeutic guidelines with clear indications for the use of antibiotics in pregnancy which have laid the foundation for the clear management of this pathology, but with multiple challenges remaining for both mother and fetus [3,4].

IE is an infection of the endocardium and predominantly affects the anatomical structures on the left side of the heart [5]. The emergence of bacterial agents resistant to standard antibiotic therapy, the increasing identification of “modern” risk factors such as intracardiac devices and intravenous drug administration and the increasing prevalence of nosocomial infections are current challenges with multiple implications for pregnant women with IE, both medically, socially and economically [6,7].

The prognosis of patients with IE depends on the age at which pregnancy is achieved, as it is known that young age is associated with a high risk of obstetric complications. Epidemiological data in the literature highlights the high mortality rate of these pathologies, both for the mother (22%) and the fetus (15%) in the absence of prompt management from multidisciplinary teams [8].

Cardiovascular risk assessment is important to perform at every pregnancy, especially in patients with a history of heart disease prior to pregnancy. The guidelines of the European Society of Cardiology [9] for the assessment of cardiovascular disease recommend the application of the World Health Organization (WHO) classification of maternal cardiovascular risk assessment, with prognostic and therapeutic implications alike. A large proportion of clinical cases reported in the literature present as complications or cause of death the occurrence of heart failure or an acute embolic cardiovascular event [10].

This article aims to review the latest information from the literature on the complex management of IE during pregnancy starting from modern risk factors, plus genetic and molecular diagnostic methods, to potential therapeutic targets contributing to a multidisciplinary and integrative approach to these cases.

## 2. Materials and Methods

We searched the electronic databases of PubMed, MEDLINE and EMBASE for clinical studies addressing the management of infective endocarditis during pregnancy, with the aim of conducting a review of recent literature on this topic, from pathophysiological mechanisms to risk factors, signs and symptoms, as well as therapeutic management, maternal and fetal outcome and future research directions.

We used the following words or phrases for searching: “infective endocarditis” or “pregnancy”, plus one of the following (in various associations): “cardiovascular risk”, “cardiovascular mortality”, “cardiovascular prognosis”, “cardiovascular risk factors”, “fetal risk”, “fetal outcome”. Observational studies, including prospective or retrospective cohort studies, RCTs, meta-analyses, guidelines and case reports related to our topic were included. All selected articles were then reviewed individually to select additional relevant publications from the reference section. Two independent reviewers selected studies by analyzing the title and abstract.

## 3. From Pathophysiology to Multidisciplinary Assessment

There are few cases reported in the literature of IE in pregnant women, which increases the importance for the academic world to understand the underlying mechanisms, the risk factors involved, the discovery of incriminating microorganisms and optimal therapeutic management, with maximum benefits for both fetus and mother.

### 3.1. Hemodynamic and Immunologic Adaptations during Pregnancy

The main physiological changes occur in the first weeks of pregnancy when some of the pregnant patients will exhibit clinical manifestations suggestive for various cardiovascular pathologies which were subclinical until that moment [11]. During pregnancy, a number of hemodynamic changes occur at the cardiovascular level predominantly affecting the hemodynamic status [12,13].

Heart rate increases on average by up to 25%, steady throughout pregnancy until the third trimester [14,15,16]. Starting from the 25th week of pregnancy, there is an increase in plasma volume on the one hand and on the other hand a 30% reduction in red blood cells which leads to dilution anemia [17,18].

The cardiac output increases as the pregnancy progresses, reaching in the third trimester 45% higher than baseline. Some patients may experience increases in cardiac output both at onset through increased stroke volume and in progression through vena cava compression [19].

Changes in blood pressure values should be carefully monitored during pregnancy to assess the hemodynamic impact on both mother and fetus [20,21]. Vascular resistance occurring in the first trimester of pregnancy will lead to a decrease in blood pressure values, especially for the systolic component where values can be up to 15 mmHg lower [11,22,23]. In a patient with right heart IE, the increased blood volume and hemodynamic changes described above create an increased pressure environment that can easily lead to pulmonary embolism. Systemic embolisms can occur in a similar way in pregnant patients with left heart IE [24].

During pregnancy a number of immunological adaptations occur to adapt to the fetus which increase the risk of infections [25,26]. During pregnancy, there are oscillations in immune status, with data in the literature showing a global suppression [27]. Different stages of pregnancy are associated with a distinct maternal immunological profile. Thus, if in the first trimester studies have shown the existence of a significant inflammatory substrate, the second trimester is characterized by a reduced inflammatory status creating a predisposition for the occurrence of infections [28]. If we corroborate these data with the presence of the risk factors mentioned above, we get a picture of a complex pathology, which requires a rapid and efficient multidisciplinary approach.

### 3.2. Demographics and Risk Factors

*Demographic Data*

The risk of developing IE for women with congenital heart disease is lower than that of men due to better dental and hand hygiene as well as lower rates of smoking and intravenous drug use [29,30,31]. Increased life expectancy and the rise in healthcare-associated infections have led to an increased incidence of IE among women, while in men studies have reported a decrease in incidents with age [32]. Gender also influences the mortality rate, with women having a higher risk than men [33]. An increased susceptibility to infections secondary to the relative immunosuppression associated with pregnancy exacerbates the negative impact of IE on both mother and fetus, both during pregnancy and in the first weeks postpartum [34].

Kebed et al. [35] conducted a systematic review based on an analysis of 72 clinical trials including 90 patients with peripartum IE. 98% of patients had native valve infections, with the most common cardiac structure involved being the mitral valve. In the antibiotic era, IE is no longer a disease of underdeveloped countries. Similar epidemiological phenomena have been reported in high income countries due to rising living standards and prophylactic antibiotic administration [36,37].

*Risk Factors*

The risk factors responsible for the development of IE in pregnant women are similar to those of the general population, the three main etiologies being intravenous drug administration, congenital heart disease and rheumatic heart disease [38,39]. 0.5% of patients with congenital heart disease develop IE during pregnancy [9].

**Figure 1:**
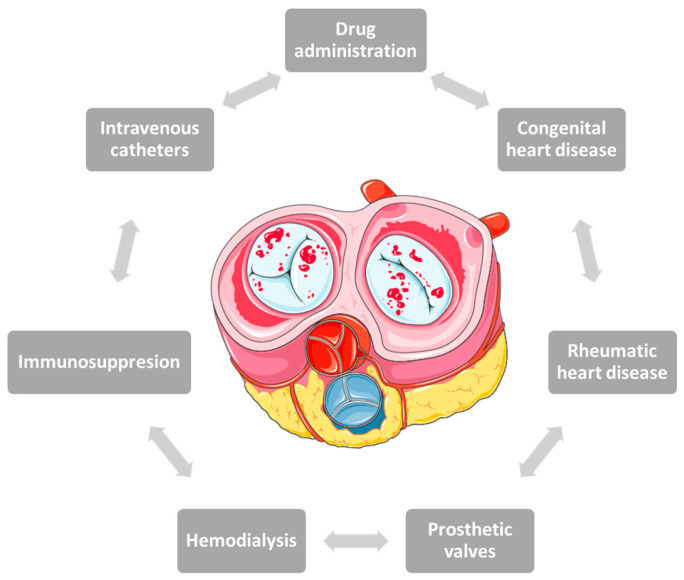
Risk factors involved in the onset of infective endocarditis in pregnancy.

In the past, the most common causes of IE in young people were rheumatic heart disease or congenital heart disease. Advances in technology have led to changes in the panel of predisposing factors over time, with age, frailty or the presence of comorbidities increasingly being blamed instead of prosthetic valves, hemodialysis, intravenous catheters or immunosuppression [40,41] (Figure 1).

Over 75% of patients who develop IE secondary to intravenous drug administration have endocardial damage, most commonly secondary to septic embolization at injection sites [42]. Tricuspid valve involvement is objectified in most cases, whereas pulmonary valve lesions are much rarer [43,44]. Isolated pulmonary valve IE is a rare entity, with few such cases reported in the literature, especially in pregnant women. Risk factors include pulmonary valve abnormalities, intravenous drugs or the presence of right heart catheters [45].

There are few cases in the literature of IE involving devices used to close atrial septal defects in pregnant patients one year after the procedure. In a case series published by Amedro et al. [46], none of the 22 patients with atrial septal defects closed with minimally invasive surgery developed IE at more than one year. This clinical finding has a pathophysiological basis in the observation that bacterial insemination occurs before neo-endothelialization of implanted devices, most commonly in the first 3 months [47].

Pregnant women may associate a partially immunocompromised status that increases their risk of developing IE in the context of the presence of comorbidities with prognostic implications on the course of pregnancy or fetal viability. This justifies the continuation of antibiotic therapy beyond the 6-month period indicated by current clinical guidelines [48,49]. The susceptibility of pregnant women to certain infectious diseases or the modulation of their severity is closely related to the placental immune response and its tropism for certain pathogens [49,50]. These rare cases present diagnostic and treatment challenges, due to the lack of accurate therapeutic recommendations. Most pregnant women have been excluded from clinical trials that have investigated the indications for percutaneous closure of atrial septal defects or patent foramen ovale [51,52,53].

Bicuspid aortic valve is the most common congenital disease, affecting 1–2% of the general population. The occurrence of IE in these patients involves the presence of a susceptible endothelium which allows for the aggregation of platelets and fibrin at this level, with the consequent formation of thrombi which represent a favorable site for the proliferation of microorganisms [54].

In the literature, there are extremely few clinical cases reported of patients with mitral valve IE requiring simultaneous valve replacement and assisted cesarean delivery. One of the main concerns in these situations is the safety of the fetus which may be jeopardized by the need for a cardiopulmonary bypass [55]. The highest risks have been reported in fetuses with a gestational age of 26 weeks and a reported survival rate of less than 28% [56].

*Etiological Agents*

Streptococci and staphylococci are the main etiological determinants of IE [57]. Staphylococci are the most common pathogens, with *Staphylococcus aureus* being an increasingly isolated pathogen that has a negative prognostic role [58,59,60]. IE can coexist with other infections, usually caused by the same pathogen. The etiological agents of left heart IE are predominantly streptococci, with staphylococci more commonly associated with right heart involvement [61]. These cases are extremely rare in pregnancy, with very few such clinical scenarios reported in the literature. One such example is a *Streptococcus oralis* infection that caused IE and meningitis simultaneously [62]. *Streptococcus oralis* causes meningitis in patients undergoing spinal anesthesia or certain dental procedures, and is rarely associated with neonatal meningitis or maternal sepsis [63].

*Bacillus cereus* is a rare pathogen responsible for the occurrence of IE in the general population, with less than 20 such clinical cases reported to date. It occurs most commonly in drug users or those with implanted cardiac devices. Shah et al. [64] isolated *Bacillus cereus* in the tricuspid valve of a 25-week pregnant patient, this being the first case of *Bacillus cereus* infection in a pregnant woman. Khafaga et al. [58] reported for the first time *Staphylococcus lugdunensis* as an etiologic agent of IE in a pregnant woman. *Staphylococcus lugdunensis* is a coagulase-negative, skin commensal staphylococcus that colonizes the perineum [65,66].

Most cases of pregnant IE reported in the literature have a bacterial infection as a microbiological substrate, with rare cases of fungi underlying this potentially fatal complication for both mother and fetus [67,68]. IE with fungal pathogens such as *Zygomycetes* are frequently nosocomial, secondary to prolonged antibiotic therapy or intravenous catheterization [69,70]. Such a clinical situation was encountered in a pregnant woman previously diagnosed with positive serology for hepatitis B [67].

### 3.3. Diagnosis and Management

The diagnostic and therapeutic management of these cases must be prompt, taking into account the maternal impact of the associated morbidity and mortality as well as the high risk of fetal death [29,35,71]. The management of IE in pregnant women must be carried out in multidisciplinary teams that individualize the therapeutic plan and identify the optimal time for surgery (in selected situations) and pregnancy termination [72]. Literature data show a reduction of in-hospital (*p* < 0.001) and one-year mortality for pregnant patients treated with multidisciplinary teams [73,74,75]. The development of such models of good practice for the benefit of patients is an ongoing concern of clinicians and researchers in the field alike, with the well-being of the mother and the fetus at the center [75,76].

*Clinical Picture*

The clinical picture of pregnant patients with IE may be the classic one with fever, vascular murmurs, petechiae and clinical signs associated with anemia and embolization [2], or may be partially masked by other symptoms associated with pregnancy. Special attention should be paid to fever in pregnancy, as it is a symptom frequently associated with various causes such as chorioamnionitis, pneumonia, various viral infections and pyelonephritis [77].

*Diagnostic Methods*

The diagnostic algorithm of pregnant patients with IE is similar to that of the pathology in the general population. Identification of the infectious agent is achieved by bacterial cultures which remain the standard test for pregnancy IE. In patients with negative cultures, direct serological testing is performed. The guidelines of the European Society of Cardiology recommend the collection of three blood cultures from different venipuncture sites, taken at an interval of at least one hour between the first and the last [31,78]. Alternatively, molecular testing (e.g., PCR testing for *Tropheryma whipplei*) or histopathological testing using resected valves can be used. The modified Duke criteria help establish the diagnosis of IE based on clinical, echocardiographic or microbiological findings [79,80].

Cell-free deoxyribonucleic acid (DNA) next-generation sequencing is a useful diagnostic tool, superior to the methods presented above because of the longer time interval in which it can identify the pathogen compared to standard blood cultures [81,82,83]. Circulating cell-free DNA was first discovered in 1948 by Mandel et al. [84,85]. Blood contains small amounts of it, predominantly from bacteria, and is a veritable reservoir of genetic material from all the body’s cells [86,87]. There are different types of cell-free DNA, the most common being circulating tumor DNA, cell-free mitochondrial DNA, cell-free fetal DNA and donor-derived cell-free DNA [88]. Recent studies have appreciated cell-free DNA as a potential clinical biomarker associated with endothelial dysfunction [89].

Cell-free DNA sequencing provides a rapid, non-invasive diagnosis, representing the only diagnostic resource in some cases of IE in which the infectious agent could not be identified by conventional microbiological identification methods [90]. In addition to diagnosis, this method can also be used for monitoring infectious pathologies or for early identification of recurrences, but further studies are needed on the decay of it in blood after treatment [91]. Decay kinetics after treatment have not been extensively reviewed in the literature to date, with few reports in the literature. Solanky et al. [91] presented the case of a 53-year-old patient diagnosed with IE involving *Bartonella quintana* at the level of the biological aortic prosthesis and periodically monitored decay kinetics after parenteral antibiotic therapy and valve resection. The group of investigators observed that after 4 weeks of parenteral antibiotic therapy, the cell-free DNA sequencing signal decreased by approximately 80%. Following excision of the aortic bioprosthesis, the decrease in cell-free DNA sequencing occurred in two phases, a rapid one in the first 24 h and a slow one occurring up to 48 h after surgery, which justifies its use as a method of monitoring the response to antibiotic therapy.

Cell-free DNA sequencing and other state-of-the-art molecular methods are showing their usefulness in the etiological diagnosis of IE, especially in those with negative bacterial cultures [92,93]. A representative example is metagenomic next-generation sequencing, which according to clinical studies published to date has a higher sensitivity than classical diagnostic methods [94,95]. Duan et al. [96] analyzed a group of 109 patients, both with and without different infectious pathologies, and demonstrated that although the sensitivity of the method is superior, no statistically significant differences were reported in specificity compared to cultures (*p* = 0.41). Comparing the infectious and non-infectious groups of patients, the investigators demonstrated that the duration of hospitalization and the 28-day death rate in the first group were statistically significant and superior. Advanced statistical techniques identified age as a determinant parameter in obtaining positive metagenomic next-generation sequencing analysis results.

**Figure 2:**
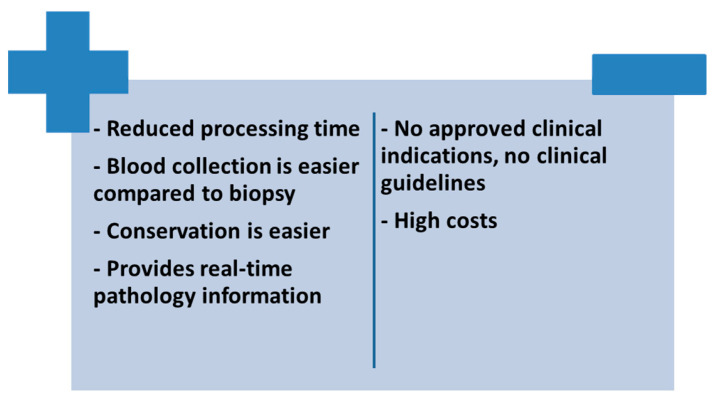
Advantages and disadvantages of using cell-free DNA sequencing in patients with IE.

Microbial cell-free DNA has a sensitivity of 89.3% and a specificity of 74.3% compared to blood cultures, with each additional day that positive results are reported associated with a 2.89-fold increased risk of metastatic infection (*p* = 0.0011) [97]. A recently published systematic review included a total of 13 clinical trials using this genetic diagnostic method in IE. Until August 2022, metagenomic next-generation sequencing has been used to identify gram positive cocci (8.9% of cases), coagulase-negative staphylococci (17.6% of cases), streptococci (37.5% of cases) and *Enterococcus faecalis* (6.6% of cases) [92].This method not only enjoys advantages such as reduced processing time, the provision of real-time information and ease of obtaining a blood sample, but also a number of disadvantages such as the high cost of the molecular extraction technique and the lack of guidelines to give this method the status of an alternative technique to conventional diagnostic methods due to the lack of large clinical studies on large groups of patients confirming the data existing so far from limited reports (Figure 2) [98].

Cell-Free DNA has also been identified in pregnant patients in the maternal circulation. In 1997, a group of investigators identified Y-specific DNA fragments in the serum and plasma of pregnant patients, representing approximately 3.4–6.2% of the plasma [99]. This discovery led over time to the use of this genetic material for prenatal aneuploidy screening. Particularly for pregnant women, the significant correlations are between the vegetation length and the serum matrix metalloproteinase-9 level and the occurrence of embolic events. Based on this observation, Thruny et al. [100] demonstrated that 64% of patients with new embolic events had vegetations greater than 10 mm in size and a serum matrix metalloproteinase-9 titer greater than 167 ng/mL [101].

*Multimodal Imaging Assessment*

**Figure 3:**
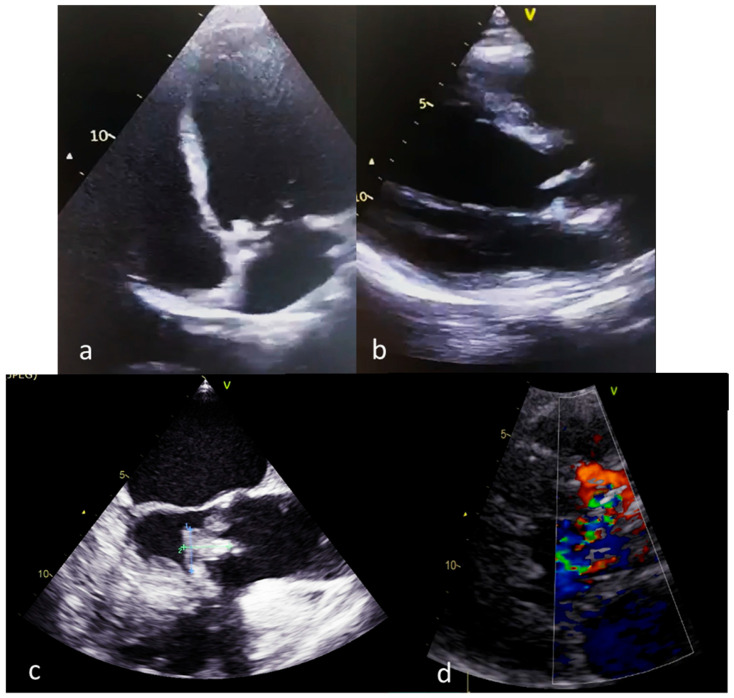
Echocardiographic findings in pregnant patients with IE (**a**,**b**): transthoracic echocardiography, aortic valve vegetations; (**c**): transesophageal echocardiography; (**d**): transthoracic echocardiography, aortic regurgitation secondary to aortic valve vegetation).

The imaging assessment of a pregnant woman with IE should focus on reducing the risk of fetal irradiation. The most commonly used methods of assessment and diagnosis are transthoracic echocardiography and nuclear magnetic resonance [77,102,103]. Echocardiographic identification of vegetations requires transesophageal echocardiography as soon as possible in patients at high risk of complications [78] (Figure 3). The CAPREG II study evaluated pregnant patients with various cardiovascular pathologies to identify predictors associated with a high risk of maternal complications such as mechanical prosthesis, high-risk associated aortic disease, pulmonary hypertension and chronic coronary syndrome [104]. Echocardiography also identifies a number of negative prognostic factors such as the presence of systolic dysfunction, the presence of a subaortic gradient of more than 30 mmHg or a pulmonary artery systolic pressure value of more than 50 mmHg in the absence of obstruction in the right ventricular outflow tract [105]. In addition to transthoracic echocardiography, transesophageal echocardiography is an additional imaging investigation required for patients with IE and mechanical valve prosthesis [106].

The persistence of the vegetation after the appearance of clinical signs of systemic embolization, plus the presence of a vegetation at the level of the anterior mitral valve of more than 10 mm, as well as the increase in the size of the vegetation at the end of the antimicrobial treatment, are echocardiographic arguments suggesting the need for surgery [107]. In terms of valve damage, a systematic review published by Kebed et al. [61] on 90 patients with peripartum IE highlights predominantly mitral valve damage in 30% of cases and then tricuspid valve damage in 25.6% of cases. The presence of IE involving the aortic valve was reported in a small number of cases (17.8%), with the percentage being half for the pulmonary valve (7.8%). The investigators of the same study highlighted concomitant damage to several valves in 12% of patients.

Valve dysfunction suggested by acute aortic or mitral regurgitation with associated signs of ventricular dysfunction or acute heart failure unresponsive to treatment along with valve perforation or rupture complete the list of indications for surgical treatment [78]. The greatest risk of embolization is for vegetations larger than 10 mm located in the anterior mitral valve [108,109]. Valvular perforations, valvular or perivalvular destructions, as well as myocardial abscesses, are echocardiographic features frequently encountered in patients with aortic bicuspid disease [110]. Lack of echocardiographic signs does not exclude IE, requiring a comprehensive, multidisciplinary interpretation of the clinical and paraclinical picture [7].

*Therapeutic Management*

Antibiotic administration must follow several principles in pregnant woman with IE and must be prompt, prolonged, personalized and combined [111]. Drug treatment of IE in pregnancy follows the principles of that given to the general population, but special attention must be paid to the fetotoxic effects of certain antibiotics [29]. Antibiotics are divided into several categories according to the safety associated with administration during pregnancy. Penicillin, ampicillin, amoxypenicillin, cephalosporins and erythromycin have no toxic effects on the fetus and can be administered to pregnant patients with IE in all trimesters according to clinical guidelines. Aminoglycosides, quinolones and tetracycline are recommended to be used in severe cases with a reserved prognosis for the mother, taking into account the associated risks. The same careful discernment is required for vancomycin, imipenem, rifampicin and teicoplanin, whose effects on the fetus cannot be excluded [9].

In pregnancy, it is recommended to delay surgery until the infection is eradicated. In high-risk situations for both mother and fetus, it is recommended to initiate antibiotic treatment for a short period of time prior to surgery despite the associated risk of pulmonary embolism [112]. Surgeries performed at a low gestational age are accompanied by low maternal risk, but fetal complications are frequently reported [113]. It is not recommended to perform surgery under 24 weeks of pregnancy, but to postpone it until 28 weeks of gestation due to the high risk of fetal death associated with insufficient growth [114]. Intravenous systems used with extracorporeal circulation for the removal of vegetation can be used as an alternative in the case of high-risk pregnancies [115].

In this population, special attention should be paid to metabolism in relation to the metabolism of administered medication, especially anesthetics [113]. From an obstetrical point of view, it is recommended to monitor the uteroplacental perfusion to maintain a mean arterial value above 70 mmHg in order to maximize its vascularity [116,117]. Pregnancy imposes a number of restrictions on the medications that can be administered, with current European guidelines recommending avoidance of renin-angiotensin-aldosterone system inhibitors and aldosterone antagonists [9,118,119]. Restrictions are also placed on inhaled anesthetics, propofol and ketamine, which stimulate neuronal apoptosis and thus negatively modulate the neuronal development of the fetus [120,121,122].

Intravenous tocolytics and antiepileptic medication are recommended during surgery to prevent the onset of labor [123]. Continuous monitoring of the fetus with cardiotocography is also recommended [2].

### 3.4. Complications and Prognostic Aspects of IE in Pregnant Women

The presence of IE in a pregnant patient, regardless of etiology, is accompanied by a high risk of short- and medium-term mortality due to multiple associated complications. Fetal viability may also be compromised in the absence of prompt management [124]. More than half of patients with IE deliver before term, at an average gestational age of 32 weeks [125]. The small number of cases presented in the literature provides little data on neonatal prognosis [126].

The most common complications that occur are congestive heart failure, perivalvular extension and systemic embolization (up to 50%) [127]. Literature data states that 50–60% of pregnant patients with IE develop some form of heart failure [128]. A recent clinical study published by Pillai et al. shows a correlation between the risk of occurrence of maternal complications (especially acute pulmonary oedema or congestive heart failure) and the risk class associated with pregnancy according to the WHO classification [124]. A recent analysis of 382 pregnant patients with IE reports cerebrovascular thrombosis (*p* < 0.001) and gastrointestinal (*p* = 0.007) and obstetric clots (*p* < 0.001) as the main postpartum complications, with negative socio-economic and medical impact [34]. Moreover, the percentage of patients where cesarean delivery was performed was higher compared to a cohort of pregnant women without an IE (56.1% vs. 2.2%) [34]. Humbert et al. [129] developed an algorithm to predict patients at high risk of cerebral embolism in which six variables were included: age, diabetes mellitus, atrial fibrillation, embolism prior to antibiotics, vegetation length and *Staphylococcus aureus* as etiologic agent. The greatest risk of embolization is in the first week after diagnosis [6,130]. Depending on the associated comorbidities, systemic embolization can occur in up to 50% of cases [65].

Mycotic aneurysms are another common complication in patients with IE, most commonly affecting the cerebral arteries in the intracranial segments [131,132]. Other complications rarely seen in pregnant patients with IE are splenic infarction and pulmonary embolism, the latter secondary to right heart IE [133,134]. Coronary embolization is a rare complication in pregnant women with IE and may be the etiological cause of silent myocardial infarction [55].

Complications in fetus are also varied and are accompanied by increased morbidity and mortality. IE during pregnancy increases the risk of stillbirth (odds ratio 2.96) or preterm birth (odds ratio 3.61) at an average gestational age of 32 weeks [34]. Premature birth is associated with a variety of fetal complications, the most common being low birth weight, low APGAR scores at birth, respiratory distress syndrome and intraventricular hemorrhage [73,135,136]. Clinical data show that about half of all newborns require prolonged hospitalization in the intensive care unit [73].

The appearance of pseudoaneurysms, fistulas and abscesses are other complications reported so far [137]. Surgical treatment becomes necessary if drug therapy is ineffective. Fetal viability also guides the need to induce labor before or during cardiac surgery [9]. Indications for surgery in pregnancy-associated IE are heart failure secondary to acute valvular regurgitation, large vegetations, sepsis or embolization [138].

The management of these cases is extremely complex, in which multidisciplinary teams must choose the best therapeutic strategy for mother and fetus alike [73]. Based on literature data, three types of clinical scenarios have been synthesized, with varying implications depending on the clinical and paraclinical data of the pregnant woman. The performance of cardiac surgery and continuation of pregnancy depends on several prognostic factors, the most important being the negative impact of cardiopulmonary bypass on the fetus and adverse effects associated with medication, especially vasoactive agents such as warfarin [55]. Vaginal or cesarean delivery followed by surgery is the second clinical scenario, which may induce worsening of heart failure in patients with hemodynamic instability. Another treatment option is to perform simultaneous caesarean section and cardiac surgery, paying special attention to the increased bleeding risk secondary to heparinization associated with cardiovascular bypass.

Cardiac surgery is also associated with a high risk for the fetus. Clinical guidelines recommend postponing cardiac surgery as much as possible until 6 weeks postpartum, and if not possible then to perform cardiac surgery in the second trimester between 13–28 weeks of pregnancy [139,140]. In some cases, the fetus may have transient or spontaneous bradycardia, which may subside at the end of surgery. As an alternative to fetal heart rate monitoring, uterine activity can be monitored. Early surgical treatment reduces the risk of embolization compared to conventional drug management by up to 25% (*p* = 0.02) [141]. Pre-pregnancy valve damage negatively influences the prognosis of pregnant women after surgery [142].

Cardiopulmonary bypass (CPB) is the biggest challenge of surgery, with literature data recommending caesarean birth prior to surgery to minimize the risk of death, which can be as high as 15% [143]. Cardiopulmonary bypass was first used in 1959 in a 6-week pregnant woman who underwent surgery to close an atrial septal defect [144]. CPB performed immediately postpartum carries a high risk of uterine bleeding, but the literature confirms that it can be performed relatively safely during pregnancy [58,145]. The main effects of cardiovascular bypass on the fetus are related to a number of factors such as timing of heparinization, infusion fluid temperature, infusion rate and associated pressure and temperature of the pregnant woman. Fetal death most commonly occurs during the cooling and rewarming periods of the bypass [39]. Maintaining the activated clotting time for more than 300 s prevents blood clots in the CPB circuit.

In the case of fetuses with a low gestational age, it is recommended to adjust cardiopulmonary bypass parameters by increasing infusion rates, maintaining normothermia and pulsatile flow and continuous monitoring of fetal heart rate [146]. The viability of the placenta is dependent on both maternal and fetal factors. Utero-placental hypoperfusion leads to bradycardia and fetal hypoxia [147]. Approximately one hour after interruption of a cardiopulmonary bypass, the fetus progressively develops respiratory acidosis secondary to increased placental vascular resistance through activation of eicosanoid products. This increase in fetal vascular resistance is poorly tolerated by the immature fetal myocardium [148,149,150].

Prematurity and stillbirth occur most frequently in urgent cases, accompanied by high surgical risk, multiple maternal co-morbidities or low gestational age [145]. Peri-operatively, administration of progesterone prevents uterine contractions and magnesium sulphate and atosiban contribute to fetal protection and maintenance of viability [140]. It is recommended as much as possible to reduce the CBP time, increase the infusion rate and continue antibiotic therapy for up to 6 weeks after surgery [151].

Prevention of massive postpartum hemorrhage events is achieved in some situations by uterine balloon tamponade, ligation of branches of the uterine artery and concomitant administration of intravenous oxytocin [151].

IE is associated with a higher risk of death for both mother and fetus. Cardiac surgery during pregnancy is associated with a high maternal mortality rate of up to 7%, regardless of the gestational age of the fetus or the practice of caesarean section before [139]. Fetal survival is superior in the case of caesarean birth prior to surgery [139]. The highest risk of death is reported in fetuses under 26 weeks gestational age [56]. Death rates vary according to the location of the lesions, being about 40% for the aortic valve, 22% for the mitral valve and 10% for the tricuspid valve [152]. There is a direct proportional relationship between maternal and fetal mortality in relation to cardiac surgery. Thus, if the risk of maternal death is minimal in the second trimester (4.6%), and doubles in the third trimester (8.8%), the risk of fetal death decreases with increasing gestational age, reaching 29% in the third trimester compared to 45% in the first weeks of pregnancy. Cesarean birth before valve replacement is accompanied by the lowest risk for the fetus (6.7%), while for the mother no significant differences were reported compared to the existing statistical data for the 3rd trimester (8.8% vs. 8.3%) [139].

## 4. Impact of COVID-19 on the Etiopathogenic Mechanisms of IE

The COVID-19 pandemic was the biggest medical, economic and human challenge of the last decade [153,154]. Since the first infections and until now, multiple clinical studies have been conducted on the impact of this virus on patients with cardiovascular disease or who are apparently healthy. These results involving both diagnostic and therapeutic roles have ensured the dynamics of therapeutic strategies. A number of specific physiological conditions, such as pregnancy, were also investigated.

Epidemiological studies have shown that SARS-COV2 infection per se has been associated with a high risk of complications in pregnant patients (regardless of gestational stage), with these complications including high risk of pre-eclampsia, premature birth and gestational diabetes [155,156]. Pregnant women are more susceptible to significant complications, with a high risk of death secondary to a viral infection due to physiological changes in the respiratory and immune systems during pregnancy and the puerperal period [157].

The link between COVID-19 and IE has been investigated in several clinical trials and two potential, independent mechanisms have been proposed [158]. Based on an animal model, Liesenborghs et al. [159] emphasize the idea that endothelial injury predisposes patients to IE secondary to *S. aureus* insemination. The same group of investigators point to a second potential mechanism, focusing on the inflammatory process in heart valves that stimulates bacterial adhesion by increasing the expression of certain structures. Rajan et al. [160] analyzed the effects of COVID-19 on pregnant women with rheumatic heart disease and observed a high risk in both groups, with no statistical significance, however, in terms of both maternal and fetal risk. The proposed mechanisms require further clinical studies to confirm or disprove the connection between the two pathologies.

## 5. Future Directions

The management of IE during pregnancy is extremely challenging in terms of the complications that can arise for both mother and fetus, as well as the limitations of medication [161]. Changing the mode of antibiotic therapy administration from intravenous to oral (with the use of two different antibiotics with different mechanisms of action) in patients with left heart IE is non-inferior according to the results of the Partial Oral Treatment of Endocarditis trial [162,163]. Overuse of antibiotics has led to the emergence of resistance in many bacterial strains, which requires clinical research to find new molecules that can be administered to pregnant women with IE [164]. One such example is daptomycin which is recommended for patients with right-sided *Staphylococcus aureus* IE, unresponsive to conventional treatment [165,166]. There are not enough clinical trials to date to allow or prohibit its use in pregnancy [167].

Clinical data on the use of ultrasound contrast agents during pregnancy are conflicting, but current recommendations are still directed towards avoiding its use as much as possible [168]. Sonobactericide non-invasively removes IE biofilm using ultrasound-activated lipid-coated microbubbles [169,170]. Recent in vitro clinical studies have demonstrated the efficacy of microbubbles against IE-associated biofilm caused by *Staphylococcus aureus* by 84% degradation [171]. In a similar study, Kouijzer et al. [172] demonstrated that vancomycin-decorated microbubbles are able to bind to the cell walls of gram-positive bacteria. However, the promising results presented above require further clinical research specifically targeting pregnant patients with IE.

Recently, the first non-antibiotic antimicrobial direct lytic agent was tested by Fowler et al. [173] in a cohort of patients with both right- and left-sided IE determined by *Staphylococcus aureus.* The investigators demonstrated that this potential therapeutic target induced a rapid bacteriolytic effect by eradicating the biofilm to which the synergistic effect associated with antibiotic therapy was added [174]. This direction of research is a promising one, with multiple therapeutic and prognostic implications among IE patients.

## 6. Conclusions

IE in pregnancy is an ongoing challenge both diagnostically and therapeutically because of the poor prognosis (for both mother and fetus) in the absence of prompt and comprehensive management. The approach to these cases must be multidisciplinary in order to decrease the risk of maternal and fetal morbidity and mortality. The development of new diagnostic methods based on molecular genetics (such as cell-free DNA) offers future prospects for the early identification of rare microbial agents encountered in everyday practice. Facilitating the opportunity for surgical intervention, decreasing maternal mortality and choosing the optimal antibiotic therapy are some of the benefits of considering cases of pregnant IE in multidisciplinary teams.

## Data Availability

Not applicable.
